# Phytochemicals in Skin Cancer Prevention and Treatment: An Updated Review

**DOI:** 10.3390/ijms19040941

**Published:** 2018-03-22

**Authors:** Chau Yee Ng, Hsi Yen, Hui-Yi Hsiao, Shih-Chi Su

**Affiliations:** 1 Department of Dermatology, Chang Gung Memorial Hospital, Linkou, Taipei, and Keelung 105, Taiwan; charlene870811@gmail.com (C.Y.N.); hsi.k.yen@gmail.com (H.Y.); ssu1@cgmh.org.tw (S.-C.S.); 2 Drug Hypersensitivity Clinical and Research Center, Chang Gung Memorial Hospital, Linkou, Taipei, and Keelung 105, Taiwan; 3 School of Medicine, College of Medicine, Chang Gung University, Taoyuan 333, Taiwan; 4 Graduate Institute of Clinical Medical Sciences, Chang Gung University, Taoyuan 333, Taiwan; 5 Center for Tissue Engineering, Chang Gung Memorial Hospital, Taoyuan 333, Taiwan; 6 Whole-Genome Research Core Laboratory of Human Diseases, Chang Gung Memorial Hospital, Keelung 204, Taiwan

**Keywords:** phytomedicine, skin cancer, chemoprevention

## Abstract

Skin is the largest human organ, our protection against various environmental assaults and noxious agents. Accumulation of these stress events may lead to the formation of skin cancers, including both melanoma and non-melanoma skin cancers. Although modern targeted therapies have ameliorated the management of cutaneous malignancies, a safer, more affordable, and more effective strategy for chemoprevention and treatment is clearly needed for the improvement of skin cancer care. Phytochemicals are biologically active compounds derived from plants and herbal products. These agents appear to be beneficial in the battle against cancer as they exert anti-carcinogenic effects and are widely available, highly tolerated, and cost-effective. Evidence has indicated that the anti-carcinogenic properties of phytochemicals are due to their anti-oxidative, anti-inflammatory, anti-proliferative, and anti-angiogenic effects. In this review, we discuss the preventive potential, therapeutic effects, bioavailability, and structure–activity relationship of these selected phytochemicals for the management of skin cancers. The knowledge compiled here will provide clues for future investigations on novel oncostatic phytochemicals and additional anti-skin cancer mechanisms.

## 1. Introduction

Skin is the largest human organ and serves as the first line protective barrier against environmental assaults. Skin barriers are especially vulnerable because they are exposed to a range of noxious agents, sun damage, and microorganisms [1]. Accumulation of these stresses may lead to skin carcinogenesis, which is a multistage process involving cancer initiation, promotion, and progression [2,3]. The initiation stage occurs after exposure to a carcinogen, such as ultraviolet (UV) radiation which causes cellular DNA damage. UV radiation induces carcinogenesis both directly, through DNA damage by photons, and indirectly, through affecting DNA, membranes, and proteins by reactive oxidative stress [4]. If DNA damage remains unrepaired, the cell undergoes irreversible permanent genetic mutations, enabling the cell with the ability for autonomous growth [5]. Followed by the initiation stage, the promotion stage occurs when these initiated cells are repeatedly exposed to compounds that promote selective clonal cell proliferation into a benign tumor over time. Regenerative proliferation, associated with repeated wounding or UV radiation, chronic inflammation, and oxidative stress, has been shown to contribute to skin tumor promotion [6]. Finally, during the progression stage, the benign tumor undergoes further genetic mutations and becomes progressively invasive, transforming into a malignant neoplasm with the ability to metastasize [2]. The exponential tumor growth during the progression stage is dependent on the recruitment of nutrition and oxygen supply through angiogenesis, a process where new blood vessels emerge from preexisting vascular structures [7].

Michael Sporn first coined the term “chemoprevention” in 1976, which now broadly refers to the use of pharmacologic or natural agents to inhibit the initiation, promotion, and progression of carcinogenesis [8]. Since then, chemoprevention has remained an area of active investigation especially with regards to the prevention of human cancer. Plants and herbal products have been used throughout history for medicinal purposes. In addition to the nutritional value derived from macro- and micronutrients, plants also contain phytochemicals, non-nutritive components, derived from the Greek “phyto” for plant [9]. Phytochemicals are biologically active compounds that may have potential health benefits, especially in the chemoprevention of cancer. Many phytochemicals have polyphenol groups consisting of multiple hydrophilic hydroxyl groups which act as scavengers for free radicals and reactive oxygen species (ROS), thereby protecting the cells from oxidative damage on DNA, protein, and lipids. Other phytochemicals exert anti-inflammatory properties by inhibiting cytokine activity or the release of inflammatory mediators, which in turn prevent the host cells from inflammation-induced damage. In addition, phytochemicals also modulate multiple cell signaling pathways and inhibit cell proliferation and angiogenesis [10,11].

**Figure 1:**
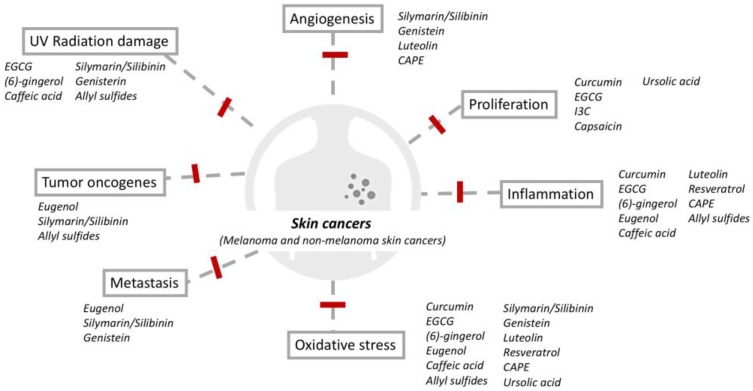
Mechanisms of phytomedicines in the chemoprevention of melanoma and non-melanoma skin cancers.

Phytochemicals have the potential to play a unique role in skin cancer. First, pre-cancerous and cancerous skin lesions are readily accessible to both the subject and physician. This is advantageous to the development of topical agents that can be applied only to the suspicious malignant area of change with minimal damage to normal skin. This contrasts with the use of phytochemical for other internal organ tumors, which may require oral ingestion of the phytochemical resulting in a systemic effect. Secondly, skin lesions and treatment efficacy can easily be evaluated by both physicians and subjects. Pathological proof is intrusive for most cancers but skin biopsies are relatively non-intrusive. Thus, future trials evaluating the effectiveness of phytochemicals in skin cancer could be more feasible. Finally, most local adverse effects can be quickly noted by subjects with topical applications; this can reduce subject discomfort and potential for long-term or more severe side effects. Several promising phytochemicals have been found in a variety of fresh fruits, vegetables, roots, and herbs, such as epigallocatechin-3-gallate, resveratrol, curcumin, proanthocyanidins, silymarin, apigenin, capsaicin, genistein, indole-3-carbinol, and luteolin; these have been considered as a means to improve cancer chemoprevention and treatment via multiple mechanisms (Figure 1). In this review, we will discuss the preventive potential, therapeutic effects, bioavailability, and structure–activity relationship of these selected phytochemicals for the management of skin cancers (Table 1).

## 2. Phytochemicals and Protective Properties against Cutaneous Carcinoma

### 2.1. Phenolic Compounds

Phenolic compounds, including polyphenols, belong to a group of common compounds widely distributed in many natural plants and herbals. More than 8000 phenolic structures are currently known, among which over 4000 flavonoids have been identified [167].

#### 2.1.1. (6)-Gingerol

(6)-Gingerol (1-[4′-hydroxy-3′-methoxyphenyl]-5-hydroxy-3-decanone) is a pungent phenol isolated from the root of the *Zingiber officinale* ginger plant, a widely used spice. In 1998, Park et al. demonstrated that topical application of (6)-gingerol on mice significantly inhibited skin papilloma formation [12]. (6)-Gingerol exerts anti-inflammatory activity by reducing epidermal ornithine decarboxylase activity, inhibiting cyclooxygenase-2 (COX-2), and suppressing nuclear factor kappa-light-chain-enhancer of activated B cells (NF-κB) via modulation of p38 mitogen-activated protein kinase (MAPK) activity [12,13]. (6)-Gingerol also exhibits anti-oxidant activity by reducing UV radiation-induced intracellular ROS levels, activation of caspase-3, -8, -9, and Fas expression [14]. Other mechanisms include activation of AP-1 DNA binding activity [15] as well as modulation of p53, Bax, Bcl-2, and survivin [16]. Although there have been no published human trials to date, there has been investigation into the incorporation of (6)-gingerol in solid lipid nanoparticles for topical use to help improve chemical stability [17]. Such a carrier system for (6)-gingerol may provide a feasible and stable option for future human investigations.

#### 2.1.2. Caffeic Acid Phenethyl Ester (CAPE)

Caffeic acid phenethyl ester (CAPE) is a major medicinal component of propolis, which is derived from honeybee products. There are several studies reporting the inhibitory effects of CAPE on many cancer types both in vitro and in vivo, including colon cancer, lung cancer, melanoma, glioma, pancreatic cancer, gastric cancer, cholangiocarcinoma, hepatocellular carcinoma, and breast cancer [18,19,20,21,22,23,24,25,26]. CAPE was shown to exhibit anti-mitogenic, anti-carcinogenic, anti-inflammatory, and immunomodulatory properties in vitro [27]. In addition, CAPE significantly inhibited the growth of mouse skin papilloma induced by the exposure to 12-*O*-tetradecanoylphorbol-13-acetate (TPA). Notably, CAPE downregulated the levels of vascular endothelial growth factor (VEGF) and multidrug resistance 1 (MDR-1), a membrane protein associated with the resistance of cancer cells to chemotherapeutic agents. CAPE also modulated cell cycle and apoptosis through NF-κB [28]. In leukemic cells, CAPE-mediated apoptosis was accompanied by upregulation of Bax, down-regulation of Bcl-2, and activation of caspase-3 [29]. At lower doses, CAPE also displayed anti-oxidant effects on mouse skin [30]. Apart from the growth of skin papilloma, there has been a lack of human clinical studies of the feasibility of CAPE for skin cancer. However, its anti-apoptotic and anti-oxidative role as well as its inhibition of vascular endothelial growth factor may act as a potential compound in the prevention of skin cancer.

#### 2.1.3. Capsaicin

Capsaicin (*trans*-8-methyl-*N*-vanillyl-6-nonenamide) is one of the most widely consumed spices worldwide. It is a phenolic acid which acts as a principal pungent component, giving red peppers such as jalapenos and red chili peppers their spiciness. There are conflicting scientific findings regarding whether capsaicin can act as a carcinogen or as a chemopreventive agent [31]. Hwang et al. showed that topical application of capsaicin promoted skin carcinogenesis in mice treated with TPA, suggesting a pro-carcinogenic effect via the activation of tyrosine kinase epidermal growth factor receptor (EGFR) and COX-2 [32]. However, Park et al. had the opposite conclusion, finding that topical application of capsaicin resulted in no significant increase in the growth of mouse skin tumors compared to controls and even moderately inhibited papilloma formation in mice [33]. Indeed, the chemopreventive activities of capsaicin have been hypothesized to be related to the induction of cell cycle arrest, apoptosis, or inhibition of cancer cell proliferation through antagonizing NF-κB, AP-1, signal transducer and activator of transcription (STAT3), and COX-2 expression [34]. Additionally, capsaicin has been observed to induce apoptosis in human cutaneous squamous cell carcinoma cell lines through inhibition of mitochondrial activity [35]. Other studies suggest that capsaicin possesses anti-migration activity on highly metastatic melanoma cells through down-regulation of phosphatidylinositol 3-kinase (PI3-K) and its downstream target, Akt [36]. Capsaicin can also have a synergistic effect on inducing apoptosis in melanoma cells lines when combined with HA14-1, an inducer of apoptosis which is a candidate for metastatic melanoma treatment [37]. It is anticipated that further investigations and epidemiological studies are required to clarify the role of capsaicin in skin carcinogenesis.

Although no studies have evaluated the use of topical capsaicin in skin cancer, we can draw from the experience of topical capsaicin in other areas. Topical capsaicin has already been used in multiple double-blind placebo-controlled trials for the treatment of chronic musculoskeletal or neuropathic pain [38]. One systematic review found that one in three patients using capsaicin had a higher rate of local adverse events such as burning, erythema, or stinging as compared to the placebo [38]. This may be an important drawback for the potential use of topical capsaicin as a chemopreventive agent in skin cancer. Thus, the development of a delivery system or formulation of capsaicin with other ingredients that can reduce the incidence of local adverse events will be an important step.

#### 2.1.4. Curcumin

Curcumin (diferuloylmethane) is a polyphenol phytochemical derived from rhizome of the golden spice turmeric (*Curcuma longa*). Numerous studies have revealed significant anti-inflammatory and anti-oxidative properties of curcumin in various inflammatory diseases, including psoriasis, ulcerative colitis, Crohn’s disease, atherosclerosis, etc. [39]. Curcumin has also shown cancer-preventive properties through the modulation of COX-2, NF-κB, 5-lipoxygenase (5-LOX), STAT3, C-reactive protein (CRP), prostaglandin E2 (PGE2), prostate-specific antigen, adhesion molecules, phosphorylase kinase, transforming growth factor-β (TGF-β), and several pro-inflammatory and apoptotic cytokines [39]. Kuttan et al. first proposed curcumin’s anti-cancer activities in humans in 1987 [40]. They found that topical curcumin could promote remarkable symptomatic relief and reduce external cancer lesion size in 62 patients. Subsequently, curcumin has been studied in various cancers either as monotherapy or in combination with other agents. It has been demonstrated that curcumin exhibits protective properties against head and neck squamous cell carcinoma, lung cancer, pancreatic cancer, colorectal cancer, prostate cancer, and multiple myeloma [39]. Dahmke et al. reported oncostatic properties of curcumin in a melanoma-bearing mouse model (C57BL/6 mouse) using B78H1 cells by enhancing the expression of miRNA-205-5p level, which plays a significant role in regulating cell proliferation and apoptosis [41]. An antiproliferative effect of curcumin against skin cancer cell line SRB12-p9 has been demonstrated in the mouse skin model. Curcumin administered by oral gavage in immunodeficient mice significantly inhibited skin squamous cell carcinoma (SCC) growth and downregulated pS6, a well-established downstream biomarker of the mammalian target of rapamycin (mTOR) and MEK/ERK pathways. Complete inhibition of SRB12-p9 cell proliferation after treatment with curcumin at a dose 20 μM or higher suggested a potent anticarcinogenic effect of curcumin in skin cancer. Both topical and oral dietary administration appears to exert similar efficacy in the mouse skin model. The safety and tolerability of curcumin is also well established by previous clinical trials, making it a potential candidate for preventive phytomedicine of skin cancer [42].

#### 2.1.5. Eugenol

Eugenol (4-allyl-2-methoxyphenol) is a phenolic component of cloves; within the human diet it can be found in aromatic spices such as nutmeg, cinnamon, bay leaves, and basil. Both topical application of eugenol and oral administration of an aqueous infusion of cloves to mice bearing skin cancer delayed and reduced the incidence of papilloma formation [43]. Eugenol exhibits anti-oxidative, anti-proliferative, and anti-inflammatory activity through a variety of mechanisms. Eugenol’s anti-oxidative property may be due to its rapid scavenging activity, inhibiting superoxide formation and lipid peroxidation [43]. Topical application of eugenol can reduce inflammation via inhibition of COX-2 and inducible nitric oxide synthase (iNOS) expression, decreasing levels of proinflammatory cytokines (IL-6, TNF-α, and PGE2), and modulating NK-κB expression [44]. Furthermore, eugenol can downregulate oncogenes, c-Myc and H-ras, modify p53 expression, and induce apoptosis by decreasing the transcription activity of E2F1 [45,46].

In a recent study, eugenol was prepared as a nanoemulsion for topical anti-inflammation use in murine skin, with a 2% eugenol formulation demonstrating better anti-inflammatory activity compared to topical piroxicam after 1.5 h [47]. However, the study noted that when piroxicam was introduced into the eugenol nanoemulsion, the anti-inflammatory effects of eugenol became non-significant, possibly through decreasing its stability and increasing its particle size. Future in vivo skin permeation studies are needed to test this hypothesis, as topical nonsteroidal anti-inflammatory drugs are widely used and its potential to negate the effect of topical eugenol is important to clarify.

### 2.2. Caffeic Acid

Caffeic acid (3,4-dihydroxycinnamic acid, CA) is one of the most abundant polyphenolic compounds, found primarily in coffee as well as several herbs, fruits, and vegetables. Bioactive CA compounds have been shown to possess anti-cancer, anti-oxidant and anti-inflammatory properties [48,49,50]. Recent studies revealed that CA inhibited tumor metastasis in colon cancer [51] and angiogenesis in renal cell carcinoma [52]. Yang et al. reported that CA significantly inhibited colony formation and EGF-induced neoplastic transformation of malignant human keratinocyte cells [53]. CA attenuated the migratory capability of cancer stem cells through an increase in p38 phophorylation and deactivation of NF-κB/snail signal pathway. Indeed, p38 decreased the DNA-binding activity of NF-κB to the promoter of snail gene which resulted in the transcriptional inactivation of snail. Further, impaired epithelial–mesenchymal transition has been observed in CA-treated malignant human keratinocytes. The epithelial–mesenchymal transition is a process by which epithelial cells lose both their cell polarity and cell–cell adhesion while gaining both migratory and invasive properties. The E-cadherin level was increased while the levels of N-cadherin and vimentin level were attenuated in CA-treated malignant human keratinocytes. Such findings indicate that CA exerts a protective effect towards skin cancer migration and invasion [47,53].

Topical application of CA to dorsal skin of the UV-induced carcinogenic mouse model was shown to suppress tumor incidence and size [47]. The MAPK pathway encompasses different signaling cascades of which the Ras-Raf-MEK-extracellular signal-regulated kinase (ERK) 1/2 pathway is one of the most commonly deregulated in human cancer. This signaling pathway mediates multiple cellular functions including cell proliferation, growth, and senescence [54]. CA directly inhibited ERK1/2 activities in vitro and exerted chemopreventive activities against solar UV-induced skin carcinogenesis [47]. In addition to MAPK, upregulation of COX-2 expression and Fyn kinase also have been detected in UVB-induced skin carcinogenesis. CA effectively suppressed UVB-induced COX-2 expression through interfering with the activity of AP-1 and NF-κB which subsequently inhibited prostaglandin E2 production as well as blocked Fyn kinase activity in a mouse model of skin carcinogenesis [55].

Chao et al. revealed that CA could provide anti-inflammatory protection by down-regulation of TNF-α, IL-6, and IL-1β mRNA and protein expression in cardiac tissue of diabetic mice [56]. Khan et al. observed that CA attenuated TPA-induced tumor progression by inhibition of oxidative stress and pro-inflammatory cytokine production [57]. Furthermore, Song et al. indicated that CA had anti-inflammatory effects by reducing the activity of myeloperoxidase (MPO) and phospholipase A2 in skin-incised mice [58]. Likewise, topical treatment with CA inhibited TPA-induced skin edema in a dose-dependent manner, leading to substantial reductions of skin thickness and tissue weight, neutrophil-mediated MPO activity, and various histopathological indicators [57]. CA also significantly reduced the mRNA and protein levels of TNF-α, IL-6, and IL-1β at the application site as well as in human keratinocytes in vitro [57]. Furthermore, CA was effective at reducing inflammatory damage induced by chronic TPA exposure. These results show that CA has anti-inflammatory activities in both acute and chronic contact dermatitis models via the blockade of inflammatory cytokine production and neutrophil-mediated MPO activity. It also can target inflammatory mediators specifically in keratinocytes. Collectively, these findings advocate the necessity of developing and testing CA for its potential use in clinics for skin cancer patients.

### 2.3. Flavonoid

Flavonoids, found in a wide range of plants, fruits, and herbs, are a group of natural compounds with variable phenolic structures. Mainly attributed to their anti-oxidative, anti-inflammatory, and anti-carcinogenic properties, these phytochemicals are well known for their beneficial effects against various diseases including skin cancer [168].

#### 2.3.1. Epigallocatechin-3-Gallate

Epigallocatechin-3-gallate (EGCG) is a polyphenol compound found in green tea. It is the most studied chemopreventive component of green tea phenols (GTP), known for its anti-oxidant, anti-inflammatory, and anti-proliferative properties [59]. Katiyar et al. have done extensive research into GTP and in 1992 they found that the anti-inflammatory properties of GTP may be associated with inhibition of COX and lipoxygenase activity, lowering skin tumor burden with decreased epidermal edema and hyperplasia [60]. They later demonstrated the anti-oxidant effect of EGCG on human skin by topical EGCG application to human skin, which reduced UV radiation-induced production of hydrogen peroxide and nitric oxide in both the epidermis and dermis [61]. Such reduction may be related to the inhibition of MAPK signaling pathways [62]. Other proposed anti-proliferative actions include modulation of NF-κB pathways [63,64], inhibition of tumor promotor-induced activator protein (AP-1) activation [65], inhibition of angiogenesis, and recruitment of cytotoxic T cells [66].

With regard to melanoma cells, Nihal et al. have shown EGCG to sensitize melanoma cells to interferon-induced growth inhibition, decreasing cell proliferation, and inducing apoptosis [67]. Interestingly, they found that the combination of EGCG with interferon was more effective than either agents alone, suggesting a synergistic role of EGCG in tumor suppression. Possible mechanisms include downregulation of inflammasome, which decreases interleukin (IL)-1β secretion and reduces NF-κB activity, leading to decreased tumor growth [68]. Recently, EGCG has also been found to inhibit melanoma cell invasion and migration by attenuating the activity of tumor necrosis factor (TNF) receptor-associated factor 6 (TRAF6) [69].

EGCG is one of the better studied phytochemicals for skin cancer chemoprevention with several small human trials. The issue of oral versus topical administration remains an important issue. One early study noted that mice given green tea constituents orally or via injection resulted in inhibition or even regression of UV-induced skin papillomas [70]. However, another study demonstrated that tumor reduction in mice was only observed by using topical application of purified EGCG but not oral administration of EGCG [71]. This discrepancy between topical and oral administration of EGCG may be accounted for by insufficient distribution of EGCG in the skin after oral ingestion. A study of human volunteers using topical green tea polyphenols confirmed the protective activities of GTP against UV radiation-induced erythema [72]. However, a recent single-blind randomized clinical trial of 50 volunteers showed that healthy adults who had oral green tea extract supplementation with vitamin C did not have significantly reduced skin erythema or leukocyte infiltration compared to the placebo group [73]. In another double-blind phase II randomized clinical trial including 51 participants with a precancerous skin disorder, actinic keratosis, EGCG was topically applied to one forearm with actinic keratosis while a placebo ointment was used on the contralateral forearm for 12 weeks. There was no significant difference observed between the two groups at the end of the study [74]. The authors hypothesized that the topical EGCG may not have been active in the formulation, possibly due to poor bioavailability. These findings suggest topical application of ECGG may be more effective than oral administration for skin cancer chemoprevention, but the optimal formulation for topical EGCG requires further investigation. Finally, another area for future studies is the potential role of EGCG as a synergistic treatment for skin cancer management.

#### 2.3.2. Genistein

Genistein (4′,5,7-trihydroxyisoflavone) is a soybean-derived isoflavone compound [75]. Diets rich in soybeans have long been used as dietary supplements for osteoporosis, cardiovascular disease, and cancers [76]. Genistein is the most abundant phytoestrogen compound in soybeans and possesses potent anti-oxidant, anti-inflammatory, and anti-proliferative effects [77,78,79]. Cancer chemopreventive properties of genistein have been demonstrated in various malignancies, including breast cancer and neuroblastoma, as well as both melanoma and non-melanoma skin cancers [11,80]. Genistein has been shown to exert anti-angiogenesis properties, reduce tumor proliferation and metastasis, induce cell cycle arrests [81], and promote cell apoptosis [82]. The administration of genistein reduced UV-induced sunburn in humans, protecting from both photoaging and UV-induced skin cancer [83]. Pretreatment of animals with genistein prior to UVB exposure inhibited UVB-induced oxidative damage in the epidermis of hairless mice through hydrogen peroxidase (H_2_O_2_) and malondialdehyde (MDA) lipid peroxidation [83]. Photoprotective properties of genistein have also been demonstrated in human reconstituted skin as genistein inhibited UVB-induced pyrimidine dimer formation in a dose-dependent manner [84]. Moreover, genistein has been shown to have beneficial effects against melanoma cells through interference with cell cycles and the inhibition of tumor growth and metastasis in a xenograft model [85,86]. Inhibition of melanoma cell cycle progression by genistein was attributed by targeting p53, p21, and a checkpoint kinase, Chk2 [87,88,89]. In addition to the regulation of cell cycle, genistein was also shown to promote melanoma cell differentiation through the stabilization of protein-linked DNA strand breakage and inhibit angiogenesis [90,91,92]. While evidence supporting the use of genistein for chemoprevention of melanoma and non-melanoma skin cancer in human reconstituted skin and cellular studies. Further clinical trials to confirm the route of administration, dosing, and proper randomized control trials are needed to confirm the efficacy of genistein in preventing skin cancer.

#### 2.3.3. Luteolin

Luteolin is a flavonoid compound found in wide variety of dietary sources, such as carrots, peppers, celery, and olives. Luteolin is known for its anti-oxidant, anti-inflammatory, and anti-tumor activities as well as its ability to inhibit angiogenesis, promote apoptosis, and sensitize cells to anti-cancer therapies in a variety of malignancies [93]. Numerous studies have found that luteolin induced melanogenesis and reduced invasive potential of melanoma cells through regulating β3 integrin/focal adhesion kinase (FAK) signal pathway [94,95]. In addition, luteolin was shown to promote apoptosis and inhibit cell growth in melanoma cells through the upregulation of Bax, downregulation of Bcl-3, and attenuation of ERK1/2 signaling [96,97]. Although previous studies of this component appear promising in terms of tumor prevention, in vivo and human studies for efficacy and drug bioavailability are still limited.

#### 2.3.4. Silymarin and Silibinin

Silibinin, also known as milk thistle, is an isolated compound from the seeds of *Silybum marianum* (L.) Gaertn (Family Asteraceae). Silibinin is the main and active component of Silymarin complex, which consists of flavanoids and flavonolignans. The use of silibinin has been limited as a result of its poor bioavailability; however, several efforts have been made to modify formulation for better absorption, such as through nanosuspensions [98]. Historically, Silymarin has been extensively used for its hepatoprotective effects; however, additional health beneficial effects have recently been recognized [99]. Most of these effects have been attributed to the direct and/or indirect anti-oxidative capacity of silymarin, such as serving as the scavenger of ROS, the scavenger of phenylglyoxylic ketyl radicals, and a chain-breaking antioxidant [100].

Several clinical studies have analyzed the chemopreventive property of silymarin on various types of cancer, including skin cancer [99]. Agarwal et al. first reported the cancer-preventive activity of silymarin through the inhibition of TPA-induced tumor promotion in mouse skin. Such silymarin-mediated inhibition was attributed to its ability to attenuate the activity and expression of epidermal ornithine decarboxylase [101]. Another study demonstrated that silibinin targeted the cyclin-dependent kinase pathway, exerting strong anti-cancer effects through the inducement of cell cycle arrest [102]. Moreover, neo-angiogenesis is an important constituent of tumor microenvironment whereby nutrients and oxygen are supplied for tumor cell growth and distant metastasis. Silibinin has been shown to antagonize angiogenesis by targeting on the VEGF receptors and iNOS [103,104]. Furthermore, through both intrinsic and extrinsic pathways, silibinin also promotes tumor apoptosis [105,106].

Katiyar et al. demonstrated the protective effects of silymarin against UVB radiation-induced skin tumor progression in a mouse model of photocarcinogenesis [107,108]. In short-term experiments, silymarin application resulted in the inhibition of UVB-induced sunburn, apoptotic cell formation, skin edema, depletion of catalase activity, and induction of ornithin decarboxylase (ODC) and COX expressions. Similar protective effects have also been observed in the use of silibinin, as early biomarkers of UVB-damaged skin, such as thymidine dimer-positive cells, proliferating cell nuclear antigen, and apoptotic sunburn cells, were reduced after treatment with silibinin [109]. The efficacy of silibinin on targeting MAPK-mediated mitogenic signaling has been reported extensively in previous studies [110,111,112]. MAPK signaling is important for cancer cell migration and invasion [113]. Both topical and dietary administration of silibinin inhibited MAPK (ERK1/2, JNK and p38) and Akt activation, induced by either acute or chronic UVB exposure in SKH-1 mouse skin [111]. Vaid et al. have also demonstrated that silymarin reduced nuclear accumulation of β-catenin in human melanoma cells and inhibited melanoma cell migration in a concentration-dependent manner [114,115]. Results suggest that silymarin/silibinin could act as a potent chemopreventive agent against skin cancer and photocarcinogenesis. Future controlled clinical trials of silymarin on skin cancer chemoprevention, focusing on the toxicity and oral bioavailability of this compound in humans, are warranted.

### 2.4. Resveratrol

Resveratrol (3,5,4′-trihydroxy-trans-stilbene) is stilbene polyphenol. It is commonly found in grapes, peanuts, mulberries, and red wine in the human diet. Topical application of resveratrol has been shown to inhibit skin tumor initiation, promotion, and progression in murine models [116,117]. Mechanisms proposed for its anti-carcinogenic effect include anti-oxidation, anti-inflammation, and anti-proliferation. Resveratrol is well known for its anti-oxidant properties [118], acting as a potent scavenger of peroxyl and superoxide radicals [119] and significantly reducing ROS free radicals in human skin fibroblast cells in vitro [120]. In addition to its anti-oxidant effects, resveratrol also antagonizes inflammation through inhibiting the activity of COX-1 in vitro [116] and COX-2 in mouse skin [121], mainly via the inhibition of NF-κB and the suppression of both ERK and p38 MAPK [118,122,123]. The anti-proliferative effect of resveratrol is multifactorial and complicated. One study suggested that prevention of UV-mediated cutaneous damage is secondary to resveratrol’s modulation of cell cycle regulatory proteins through the inhibition of MAPK pathway [124]. Other proposed mechanisms include inhibition of survivin (an anti-apoptotic protein) and downregulation of aquaporin 3 (AQP3), a water channel protein commonly overexpressed in hyperplastic epidermal disorders, through the inhibition of ERK phosphorylation [125,126].

Resveratrol may also have a clinical potential not only as a synergistic phytochemical but also as an adjuvant treatment for melanoma. Resveratrol has a synergistic effect with other phytochemicals on the suppression of tumorigenesis and the reduction of murine epidermal hyperplasia via decreased Bcl2 expression, decreased p21, and decreased COX-2 expression [127]. Resveratrol may serve as an adjuvant to chemotherapy in treating melanomas with distant metastatic disease, demonstrated in a study where resveratrol significantly decreased melanoma cell viability and enhanced the cytotoxicity of temozolomide on malignant cells [128]. Resveratrol can also inhibit the activity of redox factor-1 (Ref-1), rendering melanoma cells more sensitive to the alkylating chemotherapeutic drug dacarbazine [129]. Moreover, attenuated expression of the anti-apoptotic and proto-oncogenic protein Akt/PKB in highly invasive melanoma cells may be another mechanism through which resveratrol exerts a chemopreventive effect for melanoma [130].

Notably, oral resveratrol has been observed to have poor bioavailability in vivo due to rapid clearance by intestinal and liver metabolism, resulting in reduced systemic concentrations in the human body [118]. This may restrict its access to the skin and tumor, perhaps explaining its inability to inhibit tumor growth when given orally to mice implanted with melanoma tumors [123]. Therefore, the topical application of resveratrol may be a more feasible chemopreventive approach. Currently, topical application of cream containing resveratrol has been tested and has shown significant improvement of hydration, luminosity, and elasticity of the skin without any side effects in healthy adults [131]. In another study of 55 patients, a combination of topical resveratrol with baicalin and vitamin E resulted in improvement of photodamaged skin over 12 weeks [132]. However, these clinical studies are limited by their small sample size and investigation of non-cancerous skin. While the effectiveness and safety of topical resveratrol in human skin cancer prevention requires larger human trials, these encouraging initial results suggest exciting potential.

### 2.5. Ursolic Acid

Ursolic Acid is a terpenoid compound found in herbs such as rosemary, thyme, and basil and has been shown to possess anti-proliferative, anti-inflammatory, and anti-oxidant activities. In 1986, Tokuda et al. first reported the inhibition of tumor production from topical ursolic acid in a mouse skin model [133]. Similar results were also observed when both rosemary and its constituent ursolic acid were topically applied to skin tumor-bearing mice, resulting in a reduction in the number of tumors [134].

Potential anti-proliferative mechanisms include modulation of the cell cycle, with ursolic acid modifying the G1 phase cell cycle and altering the expression of p21WAF1, a cell cycle regulator [135,136]. Another study demonstrated that ursolic acid inhibited IκBα kinase and p65 phosphorylation, resulting in the suppression of NF-κB [137]. Such ursolic acid-mediated inhibition of NF-κB was correlated with the reduction of pro-inflammatory COX-2, cyclin D1, and matrix metalloproteinase 9 activity. Moreover, ursolic acid can induce apoptosis in melanoma cell lines by caspase-3 activation via mitochondrial intrinsic pathway, upregulating p53 and caspase-3, and downregulating Bcl-2 [138,139]. In addition to regulating cell cycle and inducing apoptosis, ursolic acid has also been shown to elicit photoprotective anti-oxidant effects in UVB-irradiated human lymphocytes as pretreatment of ursolic acid resulted in lower lipid hydroperoxide levels and improved anti-oxidant levels [140].

Although there have been no human skin cancer trials for ursolic acid to date, it has been used as a liposome-encapsulated formulation; applied to three healthy subjects, this resulted in an increase in the ceramide content of human skin [141]. However, this sample size was small and investigated non-cancerous skin. Larger scale human studies with skin cancer subjects are necessary.

#### 2.5.1. Allyl Sulfides

Allyl sulfides, including diallyl sulfide (DAS), diallyl disulfide (DADS), and diallyl trisulfide (DATS), are the major organosulfur compounds found in garlic. Current evidence links anti-oxidant, anti-inflammatory, and anti-proliferative properties of these allyl sulfides to their chemopreventive effects on skin cancer. Belman conducted one of the earliest studies on the effect of garlic derivatives, finding that topical application of garlic oil could reduce skin tumor yield and incidence in a dose-dependent manner in mouse skin [142]. Later studies also confirmed the chemopreventive effect of topical allyl sulfides, DAS and DADS, on suppressing skin tumors in murine models [143,144,145].

Various mechanisms have been proposed for the chemopreventive effect of allyl sulfides. Topical DAS was found to modulate p53 expression in mice with skin tumors [146]. DAS can effectively reduce the number of tumors and also prolong the tumor induction time via the inducement of apoptosis in mouse skin tumors [147]. Pre-treatment with topical DAS has been reported to offer significant protection against carcinogen-induced DNA strand breaks in mouse skin [148]. Additionally, DAS may elicit its chemopreventive effects through the modulation of multiple signaling pathways, such as downregulating H-ras mRNA through inhibition of oncogenic p21 expression [149], up-regulating p53 and an anti-apoptotic protein, bax, lowering the expression of survivin and Bcl-2, and modulating the expression of PI3K/Akt and MAPKs [150]. More recently, studies have shown DAS to be beneficial against UVB-induced skin tumor formation in mice through modulating pathways involving NF-κB, COX-2, PGE2, nitric oxide, and p53 [151].

Similar to DAS, a recent study has reported that topical application of dose-dependent DADS attenuated skin tumor incidence and multiplicity in mouse models of skin carcinogenesis [152]. Shan et al. revealed that DADS upregulated many anti-oxidant enzymes, including catalase, superoxide dismutase, and glutathione peroxidase. This study also indicated the ability of DADS to increase functional nuclear transcription factor, NF-E2-related factor 2 (Nrf2) in the epidermis by upregulation of p21 protein level, allowing Nrf2 to play its vital role in maintaining cellular redox homeostasis.

DATS has also been shown to significantly reduce the incidence and multiplicity of skin papilloma through the suppression of COX-2 expression by modulating JNK or Akt signaling, which attenuates DNA binding of AP-1 [153]. Melanoma cells underwent DATS-induced apoptosis via downregulation of Bcl-2 and Bcl-xl expression [154]. Similar results were seen in human basal cell carcinoma cells, where DATS triggered apoptosis by increasing p53 and Bax expression and reducing Bcl-2 and Bcl-xl expression. This suggests a role of DATS-induced endoplasmic reticulum stress in cancer cell death [155]. Such involvement of endoplasmic reticulum in apoptosis was concordant with another study which found that DAT sensitized human melanoma cells to apoptosis [156]. Recently, DATS was shown to inhibit human melanoma cell migration and invasion by reducing the expression of matrix metalloproteinase-2 (MMP-2) and MMP-9, as well as inhibiting adhesion by disrupting the integrin signal pathways [157].

With regard to the comparisons of the different allyl sulfides, one study showed that DATS exerted stronger inhibition of COX-2 expression than DADS or DAS in human embryonic cell kidney cells [158]. Moreover, DATS showed better inhibition in the growth of human melanoma and basal cell carcinoma cell lines than did DADS and DAS [159]. The study also found that allyl sulfides inhibited cancer cell growth through G2/M arrest and apoptosis, accompanied by activation of the p53 pathway in response to oxidative stress [159]. Thus, DATS may be a more effective chemopreventive phytochemical. However, no human trials have been performed and the pharmacokinetics and bioavailability of all allyl sulfides require further investigation.

#### 2.5.2. Indole-3-Carbinol

Indole-3 carbinol (I3C) is an active metabolite of glucosinolate glucobrassicin found at high concentrations in vegetables from the family Cruciferae which includes broccoli, cauliflower, and Brussels sprouts [160,161]. Cancer chemopreventive properties of I3C have been previously demonstrated in various malignancies, including the gastrointestinal tract, lung, breast, liver, cervical, and prostate cancer [162]. It has been shown that I3C promoted apoptosis in UVB-sensitized melanoma cells through the inhibition of Bcl-2 expression and down-regulation of microphthalmia-associated transcription factor (MITF) [163,164]. Moreover, I3C inhibited the proliferation of human melanoma cells through the regulation of phosphatase and tensin homolog (PTEN) degradation [165]. Dietary administration of I3C has been shown to increase the sensitivity to chemotherapy in mouse models [166]. Thus far, study of I3C has been limited to the cellular level and mouse models. These encouraging preliminary results need to be further investigated in human skin models and clinical trials to prove its effectiveness.

## 3. Conclusions

In conclusion, increasing evidence indicates that phytochemicals are important for cancer prevention and intervention. Phytochemicals may not be as effective as conventional chemotherapeutic or pharmaceutical agents, but their potential in cancer prevention is clear. The use of phytochemicals in skin cancer prevention and intervention is very attractive as these agents are widely available, cost-effective, and highly tolerated. The use of phytochemicals for skin cancer is advantageous to prove the effectiveness of phytochemical compounds as the organ is approachable with direct observation and readily accessible to topical treatment. Current literature has demonstrated the anti-carcinogenic effects of phytochemicals through regulation of multiple different signaling pathways which have been tightly involved in versatile protective actions, including anti-oxidation, anti-metastasis, anti-inflammation, anti-angiogenesis, and epigenetic/cancer stem cells modification. These natural ingredients may also potentially shield and reverse the damaging effects derived from solar UV radiation and other environmental carcinogens. Combined with the use of sunscreen, this may serve as a reasonable strategy for skin cancer prevention. As the primary barrier against environmental assaults, normal skin needs to proliferate and differentiate continuously at a relatively high pace. The antiproliferative effect of phytochemicals need to specifically target highly proliferative tumor cells to minimize potential adverse reactions to the skin. Accordingly, topical application may be an ideal route of delivery. To date, although mounting in vitro and epidemiologic evidence supports the chemoprotective efficacy of phytochemicals in skin cancer, controlled studies with blinded evaluators are still needed to further assess their protective properties, pharmacokinetics, and bioavailability in the human body (Table 2). For topical formulation, issues such as enhanced skin penetration, stability of the compounded formulation, drug concentration, and length of treatment warrant further investigation to enable translation of in vitro and murine studies to useful human clinical treatment. Furthermore, studies focusing on controlled drug release through topical or oral delivery systems as well as the interaction of phytochemicals with conventional skin cancer therapies in the complicated process of cancerization are required (Table 3).

## Figures and Tables

**Table 1:** Summary of phytochemicals and postulated mechanisms for skin cancer chemoprevention.

Category	Phytochemical	Source	Structure	Molecular Targets	Ref.
Phenolic compounds	(6)-gingerol	Ginger	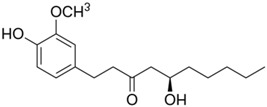	↓NF-κB, ↓p53, ↓survivin, ↓Bcl2, ↓Bax, ↓COX-2, ↓AP-1, ↓p38	[12,13,14,15,16,17]
	Caffeic acid phenethyl ester	Honey bee propolis	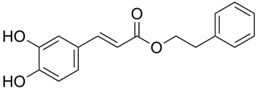	↓VEGF, ↓MDR-1, ↓NF-κB, ↑Bax, ↓Bcl-2, ↓Caspase-3	[18,19,20,21,22,23,24,25,26,27,28,29,30]
	Capsaicin	Red chili peppers, jalapenos	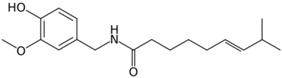	↓NF-κB, ↓AP-1, ↓STAT3, ↓PI3-K, ↓Akt, ↓COX-2	[31,32,33,34,35,36,37,38]
	Curcumin	Tumeric	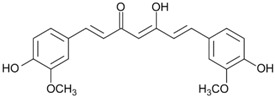	↓COX-2, ↓NF-κB, ↓5LOX, ↓STAT3, ↓CRP, ↓PGE2, ↓TGF-β	[39,40,41,42]
	Eugenol	Cloves, nutmeg, cinnamon, bay leaves, basil	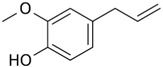	↓*c-Myc* and *H-ras*, ↓E2F1, ↓superoxide formation, ↓lipid peroxidation, ↓COX-2, ↓iNOS, ↓IL-6, ↓TNF-α, ↓PGE2	[43,44,45,46,47]
Polyphenol: phenolic acid	Caffeic acid	Coffee	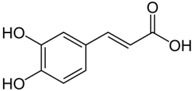	↓NF-κB, ↓AP-1, ↓MAPK, ↓COX, ↓TNF-α, IL-6, and IL-1β, ↓MPO	[48,49,50,51,52,53,54,55,56,57,58]
Polyphenol: flavonoid	Epigallocatechin-3-gallate	Green tea	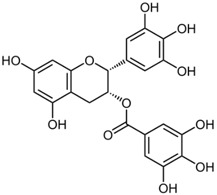	↓NF-κB, ↓AP-1, ↓IL-1β, ↓TRAF6, ↓IL-1β, ↓MAPK, ↓COX	[59,60,61,62,63,64,65,66,67,68,69,70,71,72,73,74]
	Genistein	Soybean	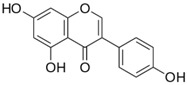	↓H_2_O_2_, ↓MDA, ↓p53, ↓p21, ↓Chk2	[75,76,77,78,79,80,81,82,83,84,85,86,87,88,89,90,91,92]
	Luteolin	Carrots, peppers, celery, oliver	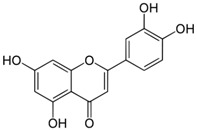	↓Bcl-3, ↑Bax, ↓ERK1/2, ↓Akt, ↓AP-1, ↓NF-κB, ↓COX-2	[93,94,95,96,97]
	Silymarin and Silibinin	Milk thistle	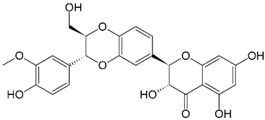	↓VEGF, iNOS, ↓MAPK family (ERK1/2, JNK and p38), ↓Akt activation	[98,99,100,101,102,103,104,105,106,107,108,109,110,111,112,113,114,115]
Polyphenol: stilbene	Resveratrol	Grapes, peanuts, mulberries, red wine	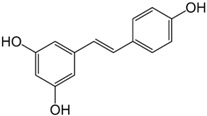	↓NF-κB, ↓ERK, ↓p38 MAP, ↓MAPK, ↓survivin, ↓Bcl2↓AQP3, ↓Akt/PKB, ↓COX-1 and COX-2	[116,117,118,119,120,121,122,123,124,125,126,127,128,129,130,131,132]
Terpenoid	Ursolic acid	Basil	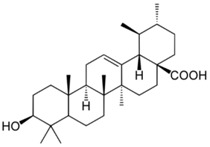	↓IκBα kinase, ↓p65, ↓NF-κB, ↑p53, ↑caspase-3, ↓Bcl-2, ↓lipid hydroperoxide, ↓COX-2	[133,134,135,136,137,138,139,140,141]
Organosulfur	Allyl sulfides	Garlic	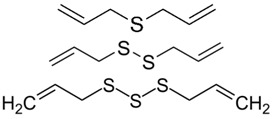	↓p21/ras, ↑p53, ↓Bcl-2, ↓NF-κB, ↑Nrf2, ↑Bax, ↓MMP-2 and MMP-9, ↓nitric oxide, ↑catalase, superoxide dismutase, and glutathione peroxidase, ↓COX-2	[142,143,144,145,146,147,148,149,150,151,152,153,154,155,156,157,158,159]
	Indole-3-carbinol	Cabbage	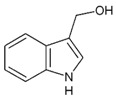	↓Bcl-2, ↓MITF	[160,161,162,163,164,165,166]

Cyclooxygenase-2 (COX-2), nuclear factor kappa-light-chain-enhancer of activated B cells (NF-κB), 5-lipoxygenase (5-LOX), phosphatidylinositol 3-kinase (PI3-K); signal transducer and activator of transcription (STAT3), prostaglandin E2 (PGE2), transforming growth factor-β (TGF-β), mitogen-activated protein kinase (MAPK), extracellular signal-regulated kinase (ERK) and p38 mitogen-activated protein (MAP) kinase, activator protein (AP-1), anti-apoptotic and proto-oncogenic protein, Akt/PKB, aquaporin 3 (AQP3), pro-apoptotic (Bax), anti-apoptotic(Bcl-2), interleukin(IL), microphthalmia-associated transcription factor (MITF), vascular endothelial growth factor (VEGF), multidrug resistance 1 (MDR-1), inducible nitric oxide synthase (iNOS), myeloperoxidase (MPO), tumor necrosis factor (TNF), epidermal growth factor (EGF), 12-*O*-tetradecanoylphorbol-13-acetate (TPA), TNF receptor-associated factor 6 (TRAF6), ornithin decarboxylase (ODC), hydrogen peroxidase (H_2_O_2_) and malondialdehyde (MDA), epidermal growth factor receptor (EGFR), 7,12-dimethylbenz[a]anthracene (DMBA), phosphatidylinositol 3-kinase (PI3-K), extracellular signal-regulated kinase (ERK), aquaporin 3 (AQP3), matrix metalloproteinase (MMP), oncogenes (*c-Myc* and *H-ras*), tumor suppressor gene (p53).

**Table 2:** Current Limitations of Phytochemicals in Skin Cancer.

Formulation and delivery systems for optimal human bioavailability remains undetermined for most phytochemicals.
Adverse effects from the use of phytochemical have been reported
Lack of evidence for recommendation of the use of phytochemical in skin cancer prevention and management

**Table 3:** Areas for Future Research.

Large controlled human trials to analyze clinical outcome measures (reduction of skin cancer incidence and skin cancer morbidity and mortality rates)
Development of formulations for optimal delivery systems and increased human bioavailability
Development of formulations combining phytochemical with other ingredients to reduce adverse effects
Combination of two or more phytochemicals in the same formulation for synergistic effect
Interaction of phytochemicals and current conventional chemotherapy

## Abbreviations

COX-2	Cyclooxygenase-2
NF-κB	Nuclear factor kappa-light-chain-enhancer of activated B cells
5-LOX	5-Lipoxygenase
STAT3	Signal transducer and activator of transcription
CRP	C-reactive protein
PGE2	Prostaglandin E2
TGF-β	Transforming growth factor-β
MAPK	Mitogen-activated protein kinase
AP-1	Activator protein
IL	Interleukin
MITF	Microphthalmia-associated transcription factor
VEGF	Vascular endothelial growth factor
iNOS	Inducible nitric oxide synthase
MPO	Myeloperoxidase
TNF	Tumor necrosis factor
EGF	Epidermal growth factor
TPA	12-*O*-tetradecanoylphorbol-13-acetate
ODC	Ornithin decarboxylase
H_2_O_2_	Hydrogen peroxidase
MDA	Malondialdehyde
EGFR	Epidermal growth factor receptor
DMBA	7,12-dimethylbenz[a]anthracene
PI3-K	Phosphatidylinositol 3-kinase
ERK	Extracellular signal-regulated kinas
AQP3	Aquaporin 3
MMP	Matrix metalloproteinase
